# First person – Mami Uemura and Mayumi Higashi

**DOI:** 10.1242/dmm.044321

**Published:** 2020-04-03

## Abstract

First Person is a series of interviews with the first authors of a selection of papers published in Disease Models & Mechanisms (DMM), helping early-career researchers promote themselves alongside their papers. Mami Uemura and Mayumi Higashi are joint first authors on ‘Gallbladder wall abnormality in biliary atresia of mouse *Sox17*^+/−^ neonates and human infants’, published in DMM. Mami is a researcher in the lab of Yoshiakira Kanai at the Department of Veterinary Anatomy, the University of Tokyo, Japan, investigating the pathologic clarification of hepatobiliary diseases such as biliary atresia. Mayumi is a researcher and pediatric surgeon in the lab of Tatsuro Tajiri at the Department of Pediatric Surgery, Kyoto Prefectural University of Medicine, Japan, investigating pediatric liver diseases and the development of therapies.


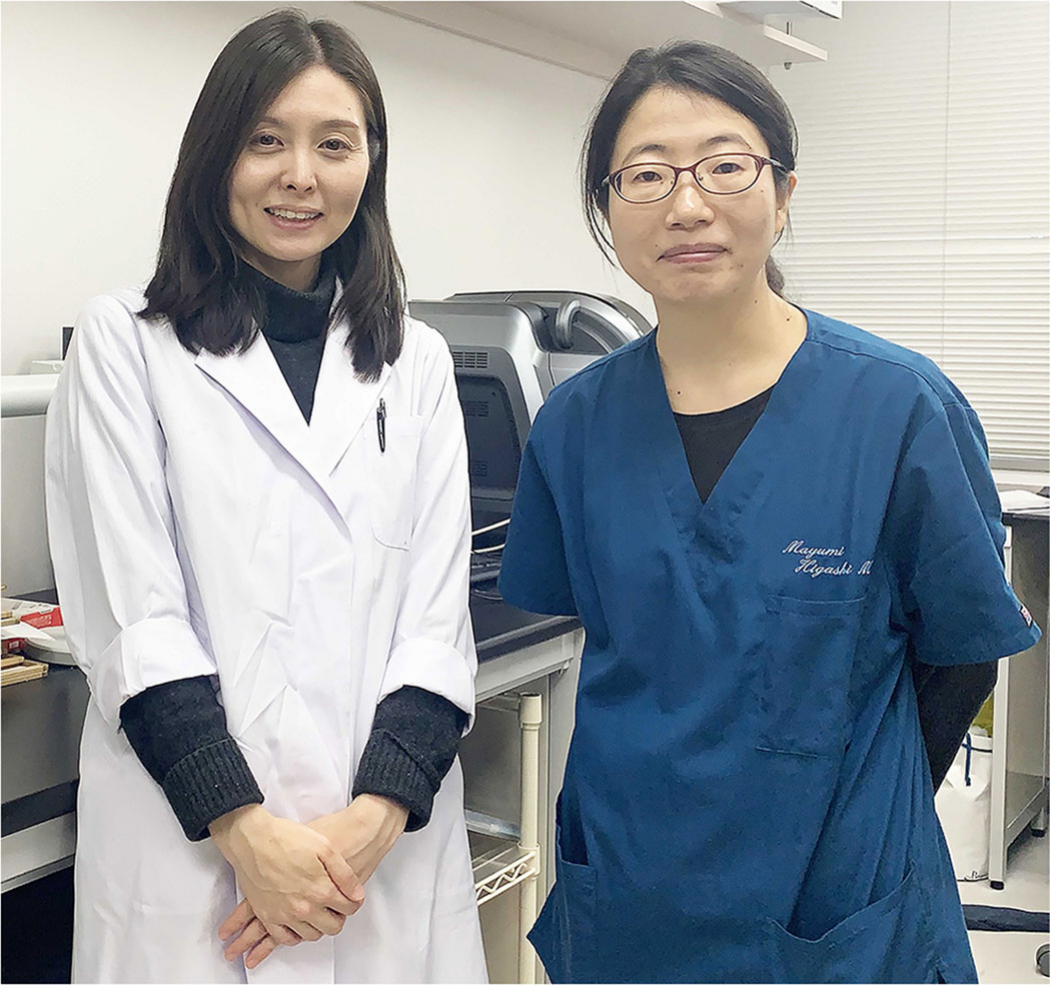


**Mami Uemura and Mayumi Higashi**

**How would you explain the main findings of your paper to non-scientific family and friends?**

MU: Biliary atresia (BA) is a disease of the liver and biliary ducts (i.e. gallbladder, cystic duct, hepatic duct and common bile duct) that develops in a baby soon after birth. BA is often regarded as an ‘orphan disease’ and it is largely unknown as to what causes and follows the onset process. We revealed that reduced *SOX17* gene expression and increased glandular structures in the BA biliary duct (gallbladder) are involved in the onset of BA. We think that these results are useful for understanding the cause and onset process of BA.

MH: BA is a rare but a very severe liver disease in children, and often the patients need liver transplantation because of cirrhosis. The cause of BA is not yet understood, but we discovered that the knockdown of the *Sox17* gene in mice results in findings similar to those seen in the biliary system of actual BA patients, such as obstruction of common bile duct, atrophic gallbladder or increased glandular structures.

**What are the potential implications of these results for your field of research?**

MU: BA presents as a cholangiopathy with neonatal icteric and pale stools. Clinically it has been thought that BA is not a single disease with a single cause. In our present study, we found that human BA gallbladder were classified into SOX17-low and SOX17-high groups, suggesting that SOX17 participated in the onset of BA, especially in the SOX17-low group. Therefore, we expect that the SOX17 index of gallbladder epithelium will be available for early diagnostic markers in a cholestatic disease, such as BA.

MH: The extrahepatic bile duct is obstructed and the gallbladder is atrophic in BA patients. SOX17 is a crucial gene for the development of extrahepatic cholangiocytes, and we found that its expression was unstable in BA gallbladders. We propose that the low expression of SOX17 contributes to the onset and the progression of BA.

**What are the main advantages and drawbacks of the model system you have used as it relates to the disease you are investigating?**

MU: We think that, in terms of the developmental context and mammal models, the *Sox17*-heterozygous (*Sox17*^+/−^) mouse we have used is an outstanding experimental model for studying the pathology of BA and therapeutic applications. On the other hand, it has been suggested that virus infection and inflammation are the main cause of BA. Humans present with with BA less than four months after birth, but it is seen in the *Sox17*^+/−^ mouse model by the neonatal stages, and mice die soon after birth. Moreover, because humans and mice differ in the sensitivity of hepatic impairment, our *Sox17*^+/−^ mouse model can display different phenotypes (e.g. normal intrahepatic bile duct and acute hepatitis) from the human BA patient. Therefore, the difference in time lag from onset and the sensitivity of hepatic cells make it difficult to more rigorous analyse BA pathology.

MH: The *Sox17*^+/−^ mouse is the first and exclusive genetic BA animal model, as far as I know. The cause of BA is not clearly identified, which does make the research of this disease quite difficult. The rotavirus infection model is known as a BA model, but this does not correspond to all the phenomena seen in human BA. The *Sox17*^+/−^ mouse sheds new light on BA research, and helps us to investigate this disease from a different perspective.

**Figure:**
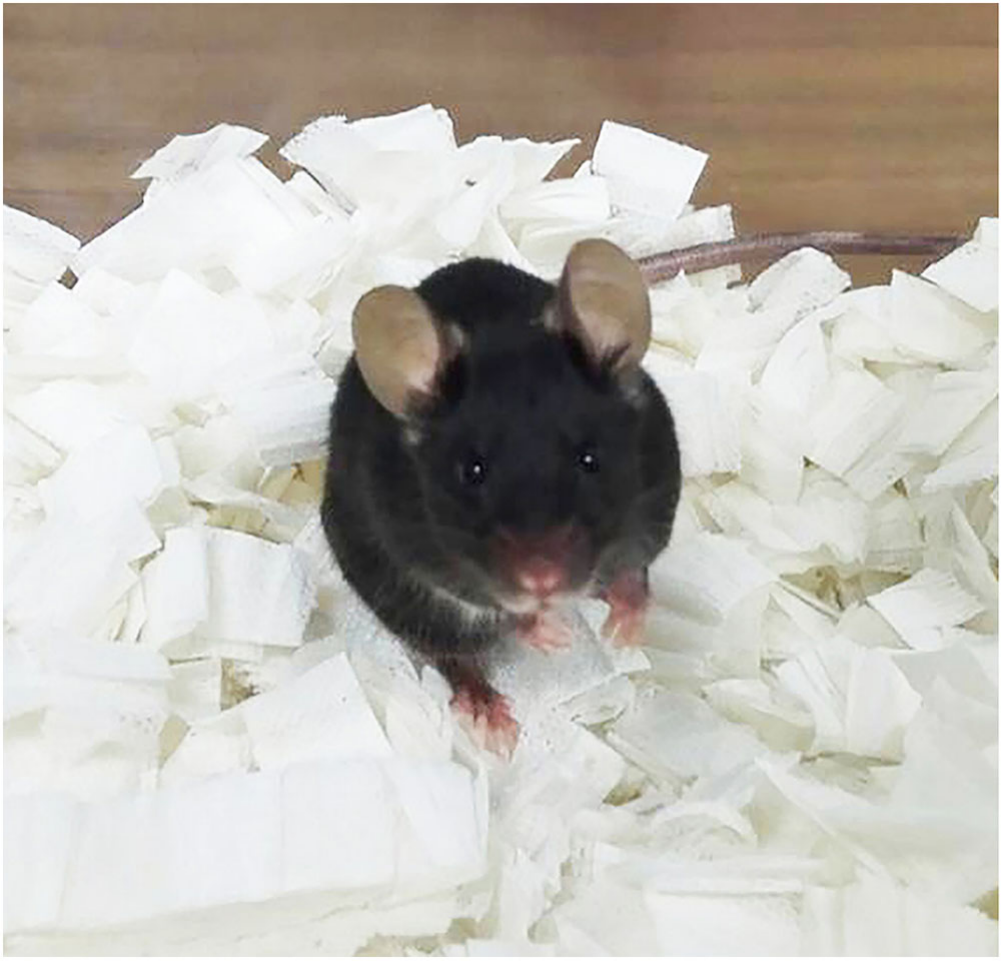
***Sox17*-heterozygous mouse is an experimental model for BA disease pathology.**

“The *Sox17*^+/−^ mouse sheds new light on BA research, and helps us to investigate this disease from a different perspective.”

**What has surprised you the most while conducting your research?**

MU: The gallbladder epithelial architecture of 13 BA cases that we used in the present research all showed a mild phenotype; 62.5% (5/8) patients exhibited reduced numbers of SOX17-positive epithelia in gallbladder walls. Unfortunately, the driver genes of BA are still unknown, but our results provide evidence that SOX17 expression maintenance in gallbladder epithelium may be one of the causes of BA onset.

MH: First of all, I was surprised when I found so many glandular structures in BA gallbladders; these were found more in the proximal region of the gallbladder and less in the distal. It was quite similar to the findings in the *Sox17*^+/−^ mouse model. Then we started to investigate the expression of SOX17 in the gallbladder of BA patients. We have not yet figured out how low SOX17 expression is related to the increase in number of glands, but it seems to be one clue to revealing the etiology of BA.

**Describe what you think is the most significant challenge impacting your research at this time and how will this be addressed over the next 10 years?**

MU: Over the past 10 years, I have researched biliary duct development and the pathology of human BA using a mouse model. In this report, I performed research using human specimens in collaboration with the pediatric surgery department for the first time and struggled with the differences between clinical research and developmental biology. From now on, I will increase my knowledge using clinical findings, and I want to investigate the mechanism and therapeutic application for common diseases between human and companion animals.

MH: It had always been difficult to conduct BA research because of its rarity and the lack of good models. Thanks to Uemura and her colleagues' *Sox17*^+/−^ mouse model, we can now try some new approaches. Not just a genetic approach, but also, for example, some immunological reactions are found in the hepatobiliary system of BA and *Sox17*^+/−^ mice – we will be able to investigate them in living mice.

**What changes do you think could improve the professional lives of early-career scientists?**

MU: In my opinion, an environment devoted to research and the presence of good mentors and collaborators is most important for young scientists. And I'm extremely thankful for the support from my mentors, research colleagues and lab members.

MH: Being modest and seeking truths. Actually it might not improve your life, but I think sometimes researchers just have to hang in there. And of course talking with other scientists will change your perspective any time.

“Talking with other scientists will change your perspective any time.”

**What's next for you?**

MU: I want to collaborate proactively with companies and scientists in different fields and expand my research to candidate genes and analytical pathology of disease in human and veterinary fields.

MH: As I'm in the position to see BA patients in person, I always want to find cures for BA. Now I would like to focus on the research of therapeutic targets to improve the liver functions of BA patients.
